# Successful treatment with atezolizumab in a haemodialysis patient with large cell neuroendocrine carcinoma

**DOI:** 10.1002/rcr2.1193

**Published:** 2023-07-19

**Authors:** Ryosuke Imai, Atsushi Kitamura

**Affiliations:** ^1^ Department of Pulmonary Medicine St. Luke's International Hospital Tokyo Japan

**Keywords:** atezolizumab, haemodialysis, immune checkpoint inhibitor, large cell neuroendocrine carcinoma, LCNEC

## Abstract

In this report, a modified regimen based on IMpower 133 (carboplatin + etoposide + atezolizumab) was administered to a patient diagnosed with large cell neuroendocrine carcinoma (LCNEC) who was concurrently undergoing haemodialysis. Adverse events led to the discontinuation of carboplatin and etoposide after the first course. Nevertheless, the patient exhibited reduction in pulmonary nodule and adrenal metastasis while receiving atezolizumab, indicating its sustained efficacy for a duration of 7 months. To the best of our knowledge, this is the first documented case demonstrating successful treatment with atezolizumab in LCNEC patients undergoing haemodialysis. Atezolizumab can be administered safely in patients undergoing dialysis and is a promising therapeutic option for dialysis patients with LCNEC.

## Graphical abstract

Atezolizumab was administered to a patient diagnosed with LCNEC who was concurrently undergoing haemodialysis. The patient achieved partial response while receiving atezolizumab alone, indicating its sustained efficacy for a duration of 7 months. Atezolizumab can be administered safely in patients undergoing dialysis and is a promising therapeutic option for dialysis patients with LCNEC.

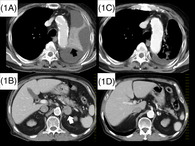

## INTRODUCTION

Large cell neuroendocrine carcinoma (LCNEC) is a rare, highly aggressive with neuroendocrine differentiation that accounts for 2%–3% of all lung cancers.^1^ The 2015 World Health Organization Classification classifies lung neuroendocrine neoplasms into four major subtypes, Typical carcinoid, atypical carcinoid, small‐cell lung cancer (SCLC) and LCNEC.^2^ Of these, SCLC and LCNEC are high‐grade neuroendocrine carcinomas and share clinical and molecular characteristics. Many studies support the efficacy of the regimen given in SCLC, that is, platinum‐etoposide, with a median progression‐free survival (mPFS) of 4.7–6.1 months.^3^ However, LCNEC has characteristic heterogeneity, and some tumours present with a neuroendocrine expression profile with a classical non‐small cell lung cancer (NSCLC) mutation; hence, treatment in the advanced stages is controversial.^4^

In addition, IMpower133 trial, a regimen of carboplatin + etoposide + atezolizumab have recently demonstrated the efficacy of combination of an immune checkpoint inhibitor (ICI)/chemotherapy for SCLC.^5^ Furthermore, the efficacy and safety of ICI for LCNEC have been reported.^6^ However, there are limited reports on the efficacy of ICI in dialysis patients with LCNEC.

Herein, we report a case of a patient receiving haemodialysis with advanced LCNEC who achieved partial response following treatment with atezolizumab.

## CASE REPORT

A septuagenarian male patient with a medical history of hypertension, tuberculous lymphadenitis, diabetes mellitus, and end‐stage renal disease receiving haemodialysis presented to our medical facility with a cough and dyspnea. Chest computed tomography (CT) scans revealed a mass in the hilar region of the left upper lobe causing obstruction of the left upper lobe branch, accompanied by enlargement of lymph nodes in stations 4 L and 11 L. A diagnosis of large cell neuroendocrine carcinoma (LCNEC) (cT2bN2M0, stage IIIA, programmed death‐ligand 1 [PD‐L1] tumour proportion score < 1%) was established through Endobronchial Ultrasound‐Guided Transbronchial Needle Aspiration, with an Eastern Cooperative Oncology Group Performance Status (ECOG‐PS) of 1.

The patient was initiated on a sequential chemoradiotherapy regimen consisting of carboplatin at a dose of 300 mg/m^2^ on day 1 and etoposide at a dose of 50 mg/m^2^ on day 1 and 3, administered prior to dialysis, every 3 weeks. This was followed by radiation therapy (60 Gy in 30 fractions for 6 weeks). During the first course of carboplatin and etoposide, the patient required hospitalization due to grade 4 neutropenia according to the Common Terminology Criteria for Adverse Events and congestive heart failure. Consequently, the carboplatin and etoposide doses were reduced by 20% starting from the second course, and pegfilgrastim was added. The chemoradiotherapy regimen was completed successfully, resulting in a complete response.

**FIGURE 1:**
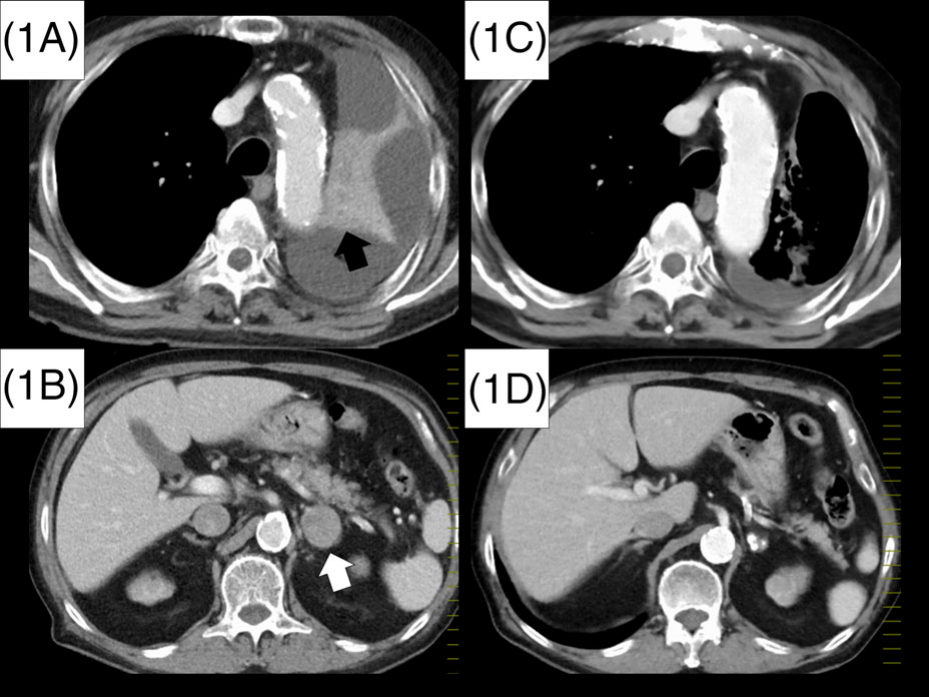
Following one course of amrubicin, a CT scan revealed an enlargement of the locally recurrent pulmonary nodule (black arrow in Figure 1A), peripheral atelectasis, and an increased size of the left adrenal lesion (white arrow in Figure 1B), indicating progressive disease. Subsequently, the administration of carboplatin, etoposide and atezolizumab was initiated. Although due to adverse events, carboplatin and etoposide were discontinued after a single course, the patient exhibited a reduction in the pulmonary nodule and adrenal metastasis while receiving atezolizumab, indicating a partial response that has been sustained for a period of 7 months (Figure 1C and D).

After 8 months of completing chemoradiotherapy, the patient developed hemoptysis. A computed tomography scan revealed a 13 mm nodule located adjacent to the leftward aortic arch, along with observed swelling of the left adrenal gland and increased F‐18 fluorodeoxyglucose accumulations on positron emission tomography‐CT. To establish a definitive diagnosis, bronchoscopy was performed, and cytology analysis of respiratory tract secretions confirmed carcinoma, diagnosing local recurrence and adrenal metastasis. Second‐line treatment with amrubicin was initiated as follows: amrubicin administered at a dose of 30 mg/m^2^, administered prior to dialysis along with pegfilgrastim on days 1–3 every 3 weeks. Following one course of treatment, a CT scan revealed an enlargement of the locally recurrent pulmonary nodule, peripheral atelectasis, and increased size of the left adrenal lesion, indicating progressive disease (Figure 1).

Considering the patient's strong preference and ECOG‐PS of 1, third‐line chemotherapy was initiated as follows: carboplatin at a dose of 240 mg/m^2^ on day 1, etoposide at a dose of 40 mg/m^2^ on day 1 and 3, atezolizumab at a dosage of 1200 mg per body on day 1, administered prior to dialysis along with pegfilgrastim on day 4 every 3 weeks. Following one course of treatment, the patient was hospitalized due to heart failure resulting from anaemia progression. Starting from the second course, only atezolizumab was continued. A CT scan performed 3 months after initiating the third‐line treatment revealed a regression of the local recurrence nodule, improvement in upper leftward atelectasis (Figure 1C), and reduction in adrenal metastasis (Figure 1D), indicating a partial response that has been maintained for 7 months.

## DISCUSSION

While the combination of ICI and chemotherapy has been widely utilized as first‐line treatment for SCLC, limited data exist regarding combination therapy in LCNEC patients. Notably, no studies have been reported thus far on the efficacy of ICI in LCNEC, particularly among individuals undergoing dialysis.

In a comprehensive study on the outcomes of ICI monotherapy for LCNEC, Sherman et al. reported on 21 cases, with an overall response rate of 33%, the complete response rate of 11%, and mPFS of 4.2 months. The study population consisted of 24% of patients with performance status 2 or greater and 90% who had received prior treatments. These findings suggest that ICI represents a promising salvage treatment option for chemo‐refractory cases with LCNEC.^6^

While there is limited data on the safety of ICIs in dialysis patients, A previous study using a large real‐world database demonstrated that the incidences of irAEs were comparable between end‐stage renal disease (ESRD) and non‐ESRD groups in patients diagnosed with lung, renal cell, bladder, head/neck cancer or melanoma treated with pembrolizumab or nivolumab.^7^ ICIs exhibit prolonged half‐lives and are excreted via receptor‐mediated endocytosis or pinocytosis facilitated by the endothelial reticulum system.^7^ Moreover, the metabolism of ICIs remains unaffected by dialysis due to their substantial molecular sizes (atezolizumab: 145 kDa).^8^ Considering pharmacokinetics, it is reasonable to infer that the utilization of ICIs in dialysis patients is generally well‐tolerated.

In the present case, following one course of carboplatin, etoposide and atezolizumab, the patient continued treatment with atezolizumab alone due to adverse events but maintained a partial response for 7 months. Although PD‐L1 is generally known as a predictive biomarker of response to ICI, the present case had <1% PD‐L1 expression. The previous data indicate that PD‐L1 expression is limited in LCNEC cases (10%–20%), whereas both SCLC and NSCLC components exhibit high tumour mutation burden (TMB) compared to conventional NSCLC and SCLC. This suggests that LCNEC may exhibit sensitivity to ICIs not solely driven by the level of PD‐L1 expression, but rather primarily associated with high TMB.^9^ Regarding safety, the patient was administered atezolizumab every 3 weeks on a non‐dialysis day, and no adverse events were observed.

In conclusion, atezolizumab demonstrated both efficacy and safety in LCNEC patients undergoing haemodialysis. Atezolizumab can be administered safely in patients undergoing dialysis and a viable option for dialysis patients with LCNEC.

## AUTHOR CONTRIBUTIONS

Ryosuke Imai and Atsushi Kitamura managed the patient, with RI taking the lead in writing the manuscript, while Atsushi Kitamura provided critical revisions.

## CONFLICT OF INTEREST STATEMENT

None declared.

## ETHICS STATEMENT

The authors declare that appropriate written informed consent was obtained for the publication of this manuscript and accompanying images.

## Data Availability

The data used to support the findings of this case report are available from the corresponding author upon reasonable request. Due to privacy and ethical considerations, some restrictions may apply to the availability of patient‐specific data.
